# Pulsatilla decoction suppresses matrix metalloproteinase-7-mediated leukocyte recruitment in dextran sulfate sodium-induced colitis mouse model

**DOI:** 10.1186/s12906-022-03696-w

**Published:** 2022-08-06

**Authors:** Ming-Kuem Lin, Ya-Ting Yang, Li-Jen Lin, Wei-Hsuan Yu, Huan-Yuan Chen

**Affiliations:** 1 Department of Chinese Pharmaceutical Sciences and Chinese Medicine Resources, College of Chinese Medicine, China Medical University, 91 Hsueh-Shih Road, Taichung, 40402 Taiwan; 2 Institute of Biomedical Sciences, Academia Sinica, 128 Academia Road, Section 2, Nankang, Taipei 115 Taiwan; 3 Institute of Biochemistry and Molecular Biology, College of Medicine, National Taiwan University, No.1 Jen Ai road section 1, Taipei, 10051 Taiwan; 4 School of Chinese Medicine, College of Chinese Medicine, China Medical University, 91 Hsueh-Shih Road, Taichung, 40402 Taiwan

**Keywords:** Pulsatilla decoction, Intestinal inflammation, Leukocyte recruitment, Matrix metalloproteinase-7, Ulcerative colitis

## Abstract

**Background:**

Intestinal inflammation is considered to be an important characteristic of ulcerative colitis (UC) and the current medical treatments for UC are usually proposed to suppress abnormal intestinal immune responses. Pulsatilla decoction (PD), a traditional Chinese medicine, is frequently used in UC treatments in Asian countries; however, the mechanism of the action of PD remains unclear. In the present study, the mechanism of the action of PD was elucidated in the dextran sulfate sodium (DSS)-induced colitis mouse model, a model to mimic UC.

**Methods:**

Murine colitis was evaluated by comparing the disease activity index score. The intestinal inflammation was examined by histology analyses. The leukocyte infiltration in the colonic tissues was examined by immunohistochemistry analyses. The cytokines level in colonic tissues was examined by Multi-Plex immunoassay. The epithelial proliferation was evaluated by histological analyses. Immunofluorescence double staining was used to examine the expression of MMP-7 in the immune cells.

**Results:**

In the DSS-induced colitis mouse model, administration of PD attenuated the intestinal inflammation, with a marked decrease in colonic infiltration of innate immune cells. Immunohistochemical analyses further showed that matrix metalloproteinase-7 (MMP-7) expressed by the infiltrating leukocytes, including neutrophils and macrophages was inhibited by PD treatment. PD increases the cytokine level of IL-6 in colonic tissues.

**Conclusion:**

PD suppresses intestinal inflammation, with a marked decrease in colonic infiltration of innate immune cells, through decreasing MMP-7 expression.

**Supplementary Information:**

The online version contains supplementary material available at 10.1186/s12906-022-03696-w.

## Introduction

Inflammatory bowel disease (IBD), which comprises two main disorders: ulcerative colitis (UC) and Crohn’s disease (CD), is characterized by chronic inflammation of the gastrointestinal tract. In general, the inflammation occurs mainly in the colon in UC, while the inflammation can affect any part of the gastrointestinal tract in CD. Multiple factors, including genetic [1], microbial [2, 3], immunological [4, 5], and environmental factors [6], have been identified to be associated with IBD; however, the exact cause of IBD remains is still uncertain. The integrity of the intestinal epithelium, a physical barrier separating luminal bacteria and mucosal immune cells, plays an important role in preventing IBD flares; however, it is often disrupted in IBD [7]. An impaired intestinal barrier leads to an increase in invading bacteria, which further exacerbates intestinal inflammation. Thus, although there is a variety of possible triggers contributing to IBD, dysregulation of the intestinal barrier seems to play a critical role in the pathogenesis of IBD.

Dextran sulfate sodium (DSS), a sulfated polysaccharide, is a chemical frequently used to induce colitis in a murine model [8]. DSS is toxic to the colonic epithelium and administration of DSS in drinking water disrupts the integrity of the intestinal epithelial barrier and consequently triggers intestinal inflammation in mice [9]. Moreover, recent transcriptome analysis has observed conserved pathways related to neutrophil chemotaxis and degranulation as well as the inflammatory immune response between DSS-induced colitis mice and UC patients [10]. Therefore, the observation of conserved inflammatory pathways between the DSS-induced colitis mouse model and UC provides a reasonable basis for using the DSS-induced colitis mouse model to investigate the regulation of intestinal inflammation. In addition, DSS-induced colitis also develops in the absence of adaptive immunity [11], highlighting the contribution of innate immune cells, such as neutrophils and macrophages, in the immunoregulation of colitis.

Matrix metalloproteinases (MMPs) are a family of zinc-containing endopeptidases. MMPs have been well-known for degrading protein components of the extracellular matrix. However, recent studies have indicated the role of MMP-7 in the regulation of inflammation and innate immunity [12, 13]. For instance, MMP-7, also known as matrilysin, can generate a transepithelial chemokine gradient and control the neutrophil efflux by shedding an epithelial cell surface proteoglycan in acute injury [14, 15]. Intestinal inflammation often occurs with intestinal tissue injury and among all leukocytes, neutrophils are the immune cells recruited to the sites of inflammation in the early response [16]. However, in the DSS-induced colitis mouse model, the recruitment of neutrophils is markedly delayed in MMP-7^−/−^ mice [17]. The delayed neutrophil recruitment further affects their infiltrating capacity and tissue locations in colonic tissues. In MMP-7^−/−^ mice, the neutrophil infiltration is mostly confined to the submucosa of the colon while in wild-type mice, the neutrophil infiltration is dispersed over both mucosa and submucosa of the colon. In addition, MMP-7 is expressed by various cells, including glandular epithelial cells, keratinocytes, fibroblasts, and macrophages. A recent transcriptomic report comparing gene expression profiles between DSS-induced colitis mice and UC patients has revealed that MMP-7 is differentially expressed in both DSS-induced colitis and UC [10], suggesting the importance of MMP-7 in UC. Moreover, MMP-7 has been found significantly upregulated in both mRNA and protein levels in inflamed colonic tissues of UC patients [18, 19]. The immunohistochemical (IHC) staining of colonic tissues from UC patients also shows a strong correlation between MMP-7 expression and the severity of UC [20, 21]. In IHC staining, MMP-7 is expressed more predominantly in inflammatory infiltrating leukocytes than in glandular epithelium, emphasizing its importance in intestinal inflammation. Together, these studies suggest that the regulation of neutrophil influx by MMP-7 proteolytic activities might critically underlie intestinal inflammation in UC.

Currently, there are multiple medical treatments available for patients with UC, including 5-aminosalicylic acid (5-ASA), corticosteroids, immunomodulators, and biological drugs [22]. The choice of which medical treatment to be administered usually depends on the severity of UC and the duration of treatments. Nevertheless, due to the high relapse rate of UC, long-term administration of medical treatments is often required [23, 24]. As a result, medical treatments with low toxicity and few side effects have become more important in treating UC. Traditional Chinese medicine (TCM), a developed branch of complementary and alternative medicine, has been practiced for over 2,000 years in Asian countries and has shown promising therapeutic effects in IBD therapy [25–28]. Pulsatilla decoction (PD), a common prescription in TCM, has been widely used in UC treatments in China [29]. PD is composed of four herbal materials, namely, Pulsatillae Radix (Bai Tou Weng, *Pulsatilla chinensis* (Bunge) Regel), Phellodendri Cortex (Huang Bai, *Phellodendron amurense* Rupr.), Coptidis Rhizoma (Huang Lian, *Coptis chinensis* Franch.), Fraxini Cortex (Qin Pi, *Fraxinus chinensis* Roxb.); these herbal materials have been found to exhibit anti-bacterial [30, 31], anti-fungal [32], anti-oxidant [33], anti-tumor [34–36] and anti-inflammatory effects [37–40]. A recent report indicates that PD is able to regulate the balance of intestinal microflora, and thus relieve infectious diarrhea [41]. Furthermore, in oxazolone-induced colitis, PD, along with three other herbal materials, Sanchi, Paeoniae Radix Rubra, and Glycyrrhizae Radix, not only suppresses the colonic inflammation but also restores the expression of epithelial tight junction proteins [42]. Despite the above research providing mechanisms of modified PD in treating oxazolone-induced colitis, the molecular mechanism of PD alone in experimental colitis is not well understood.

In the present study, we elucidate the mechanism of action of PD in a DSS-induced colitis mouse model and identify MMP-7 as an important regulator of leukocyte recruitment in murine colitis. PD down-regulates the expression level of MMP-7, accompanied by a decrease in the infiltration of innate immune cells, which suppresses colonic inflammation and consequently, ameliorates colitis. These results suggest that the MMP-7 inhibitor could be an effective treatment for gut inflammation and might be a promising therapy for IBD patients.

## Materials and methods

### Preparation of pulsatilla decoction (PD)

PD consists of four herbal materials, 10 g Pulsatillae Radix (Bai Tou Weng, *Pulsatilla chinensis* (Bge.) Regel), 15 g Phellodendri Cortex (Huang Bai, *Phellodendron amurense* Rupr.), 15 g Coptidis Rhizoma (Huang Lian, *Coptis chinensis* Franch.), 15 g Fraxini Cortex (Qin Pi, *Fraxinus chinensis* Roxb.). These four herbal materials were purchased from a traditional Chinese medicine store in Taichung, Taiwan. A total of 55 g of four herbal mixtures were added with 700 ml distilled water and decocted for 90 min. The herbal mixture solution was concentrated by rotary evaporation at 42 °C and collected by vacuum freeze-drying. PD extracts were protected from light and stored at 4 °C for future use. The identification of chemical constituents of the PD extract was performed by LC-M/MS analysis. The chemical constituents identified in the PD were listed in Supplemental Table 1.

### DSS-induced colitis mouse models

All 8-week male C57BL/6 mice were purchased from the National Laboratory Animal Center and maintained in a specific pathogen-free condition in the Academia Sinica animal facility. All experimental protocols were approved by the Institutional Animal Care and Use Committee of Academia Sinica (Protocol ID: 19–11-1362). All methods were carried out by relevant guidelines and regulations. All methods are reported by ARRIVE guidelines (https://arriveguidelines.org) for the reporting of animal experiments. Following a standard protocol of DSS (MW 36.000–50.000, MP Biomedicals)-induced colitis model, mice were given 2.5% DSS through drinking water daily until sacrifice. To investigate the pharmaceutical effects of PD, 8-week male C57BL/6 mice were randomly placed into four groups (*n* = 5 per group): control, DSS, DSS + 150 mg/kg PD, and DSS + 300 mg/kg PD. Pharmaceutical treatments started after 4 days of DSS induction and were administrated daily to mice by oral gavage. On day 8, mice were sacrificed and colonic tissues were harvested.

### Assessment of colitis

Bodyweight, stool consistency, and the presence of fecal blood in mice were measured daily during the DSS-induction period. The measurements were used to calculate the disease activity index (DAI) as described in Table 1 [43]. At the end of the experiment, the colonic tissues were trimmed from the ileocecal junction to the end of the distal colon and photographed. Colon length was measured from the beginning of the proximal colon to the end of the distal colon [44].

**Table 1:** Scoring of disease activity index (DAI)

Score	Weight loss	Stool consistency	Presence of fecal blood
0	None	Normal	Normal
1	1–5%	-	-
2	5–10%	Loose stools	Slight bleeding
3	10–20%	-	-
4	> 20%	Diarrhea	Gross bleeding

DAI = (score of weight loss) + (score of stool consistency) + (score of fecal blood)

### Histopathological analysis

Colon Swiss rolls were fixed in 10% formalin and embedded in paraffin, sectioned, and stained with hematoxylin and eosin (H&E). To evaluate the colonic inflammation, each H&E-stained colonic section was photographed at four randomly chosen regions and scored blindly using a previously published score system [45]. Briefly, two histological sub-scores were evaluated: intestinal inflammation (scale of 0–3; 0, absent; 1, mild; 2, moderate; 3, marked) and intestinal damage (scale of 0–3; 0, absent; 1, focal erosions; 2, erosions and focal ulcerations; 3, extended ulcerations). The histology score represents the sum of both sub-scores, as described in Table 2.

**Table 2:** Scoring scheme for histology score

Inflammatory cell infiltrate		Score	Intestinal architecture		Score
Severity	Extent		Epithelial changes	Mucosal architecture	
Mild	Mucosa	1	Foal erosions		1
Moderate	Mucosa and submucosa	2	Erosions	± Focal ulcerations	2
Marked	Transmural	3		Extended ulcerations ± granulation ± pseudopolyps	3

### Immunohistochemical (IHC) analysis

Proximal colonic sections (Supplemental Fig. 1) were deparaffinized and rehydrated according to the standard protocol. Antigen retrieval was performed with pH 6.0 Lab Vision™ Citrate Buffer (AP-9003–500, Thermo Fisher Scientific) constantly heating at 98 °C for 10 min in a pressure cooker. After cooled down, sections were incubated with Lab Vision™ Hydrogen Peroxide Block (TA-125-H2O2Q, Thermo Fisher Scientific) for 15 min. Sections were then washed with phosphate-buffered saline (IB3012, Omics Bio) containing 0.1% Tween® 20 (P1379, Sigma) (PBST) two times, and blocked with UltraVision Protein Block (TA-125-PBQ, Thermo Fisher Scientific) for 10 min. Next, sections were incubated with primary antibodies: rabbit anti-MMP7 (RM7C [15]) (1:100), rat anti-F4/80 (1:1000, MCA497GA, Bio-Rad), rat anti-Ly6G (1:100, Ab25377, Abcam) at 4 °C overnight. After PBST washes, sections were incubated with a secondary antibody: *N*-Histofine® Simple Stain Mouse MAX PO anti-rabbit (414341F, Nichirei Bioscience) and *N*-Histofine® Simple Stain Mouse MAX PO anti-rat (414311F, Nichirei Bioscience), respectively at room temperature for 30 min. Sections were then washed, incubated with ImmPACT DAB (SK-4105, Vector Laboratories), and counterstained with hematoxylin. All IHC quantification was performed under 20X magnification in three random fields. Quantification of Ly6G, F4/80, and Ki67 positive cells was processed through ImageJ Fiji software while the epithelial MMP-7 H-score was calculated using the DensitoQuant module in 3DHISTECH software.

### Multi-Plex immunoassay

Frozen colonic tissues were homogenized in 200 μl RIPA lysis buffer (RB4475, Omics Bio) supplemented with a protease inhibitor cocktail (HY-K0010P-10–100, MedChemExpress). The level of mIL-1β, mIL-6, mIL-10, mIL-22, mTNF-α, and mIP-10 of colonic lysates were measured in triplicates. In brief, the protein concentration of colonic lysates was determined and diluted to a final concentration of 1 mg/ml. Antibody-coupled Bio-Plex Pro™ magnetic COOH beads were incubated with 50 μl standard or diluted lysate samples for 2 h. After washes, the beads were incubated with a 35 μl detection antibody for one hour, washed, and subsequently incubated with 50 μl 1 ug/ml streptavidin–phycoerythrin (SA-PE) for 30 min. The beads were washed again, suspended with 100 μl assay buffer, and analyzed through Bio-Plex® 200 system (Bio-Rad). All assays were protected from light and performed at room temperature.

### Immunofluorescence staining

Colonic sections were deparaffinized and rehydrated according to the standard protocol. Antigen retrieval was performed with pH 6.0 Lab Vision™ Citrate Buffer (AP-9003–500, Thermo Fisher Scientific) constantly heating at 98 °C for 10 min in a pressure cooker. After cooled down, sections were incubated with Lab Vision™ Hydrogen Peroxide Block (TA-125-H2O2Q, Thermo Fisher Scientific) for 15 min. Sections were then washed with phosphate-buffered saline (IB3012, Omics Bio) containing 0.1% Tween® 20 (P1379, Sigma) (PBST) two times, and blocked with UltraVision Protein Block (TA-125-PBQ, Thermo Fisher Scientific) for 10 min. Next, sections were incubated with 50 μl primary antibodies: rabbit anti-MMP7 (1:200, RM7C [15]) and rat anti-CD45 (1:100, sc-53665, Santa Cruz), rat anti-Ly6G (1:100, Ab25377, Abcam), or rat anti-F4/80 (1:200, MCA497GA, Bio-Rad) at 4 °C overnight. After PBST washes, sections were incubated with a secondary antibody donkey anti-rabbit IgG-Alexa Flour 647 (A-31573, Invitrogen) and goat anti-rat IgG-Alexa Flour 488 (A-11006, Invitrogen) at room temperature for 30 min. After PBST washes, the slides were mounted with DAPI Fluoromount-G® (SouthernBiotech). Images were collected using Carl Zeiss LSM 510 laser scanning microscope. Quantification of MMP-7^+^ CD45^+^ immune cells, MMP-7^+^ Ly6G^+^ neutrophils, and MMP-7^+^ F4/80^+^ macrophages were performed under 40X magnification in three random fields per mouse.

### Statistical analysis

Statistical analyses were performed with GraphPad Prism software version 8.2.1. Data are expressed as mean ± SEM. For experiments compared with two groups, unpaired Student’s t-tests were used to evaluate statistical differences. When comparing multiple groups, One-way ANOVA was used for data analysis. *p*-value < 0.05 was considered significant.

## Results

### Pulsatilla decoction (PD) ameliorates DSS-induced murine colitis

**Fig. 1:**
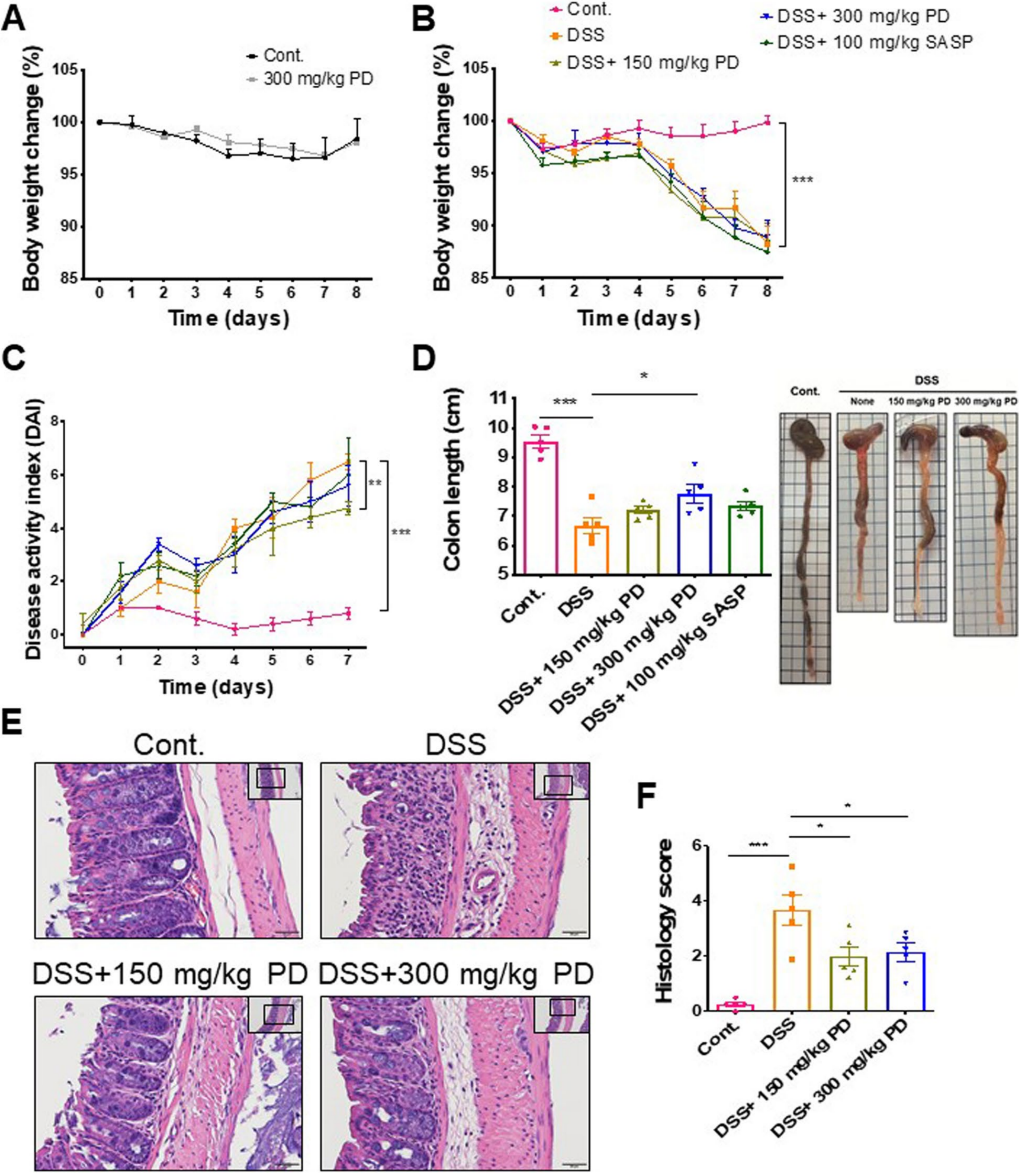
Effects of Pulsatilla decoction (PD) in DSS-induced colitis mouse model. 8-week male C57BL/6 mice were untreated (Cont.) or given with 2.5% DSS in drinking water for 8 days. Daily oral administration of PD (150 mg/kg and 300 mg/kg) were started on day 4. (**A** and **B**) Body weights were monitored daily and presented as a percentage of initial weight. (**C**) Disease activity index (DAI) scores were calculated as described in Table 1. (**D**) Colon lengths were measured on day 8 after sacrifice. The representative images of the colon of each experimental group were shown on the right. (**E**) The images of colon sections from healthy (Cont.) and DSS-exposed mice treated with or without PD (150 mg/kg and 300 mg/kg) were stained with hematoxylin and eosin (H&E). Images were taken at 20X magnification (scale bar: 50 μm, shown in the upper right) and 40X magnification (scale bar: 20 μm). (**F**) Histology scores of colon sections were calculated as described in Table 2. Data are represented as mean ± SEM, *n* = 5 mice. *p < 0.05, **p < 0.01, ***p < 0.001. PD, Pulsatilla decoction

PD is a common traditional Chinese medicine (TCM) that has been provided for UC patients in Asia for many years. However, the exact mechanism of PD itself in UC treatments remains uncertain. Thus, to understand the mechanism of PD in treating colitis, mice were first administered 2.5% DSS to induce colitis, then fed with PD. Compared to control mice, significant body weight loss, elevated disease activity index (DAI) score, and colon length shortening were observed in DSS-induced colitis mice (Figs. 1B–D). Consistent with these findings, DSS-induced colitis mice also showed a higher histology score, as reflected by increased crypt disruption, epithelial erosion and ulceration, and extensive inflammatory cell infiltration (Figs. 1E and F). After administration of PD treatments, the bodyweight loss was not significantly improved compared to the DSS-exposed mice (Fig. 1B). However, a pilot test did not show a significant difference in body weights between control and 300 mg/kg PD-administered mice, suggesting that PD alone does not affect the body weights of mice (Fig. 1A). Apart from the body weights, we also examined other characteristics of the DSS-induced colitis mouse model in PD-administered mice. The results showed that the lower dose, 150 mg/kg, but not the higher dose, 300 mg/kg, of PD, reduced the DSS-induced DAI score (Fig. 1C); yet, the higher dose, 300 mg/kg, but not lower dose, 150 mg/kg, of PD, reduced the DSS-induced colon length shortening (Fig. 1D). However, histology analysis revealed mild colonic inflammation and improved epithelial integrity in both doses of PD treatments (Figs. 1E and F). These results suggest that despite without a clear dose-dependency, PD still ameliorates DSS-induced colitis.

### PD reduces the neutrophil and macrophage infiltration in colonic inflammation

**Fig. 2:**
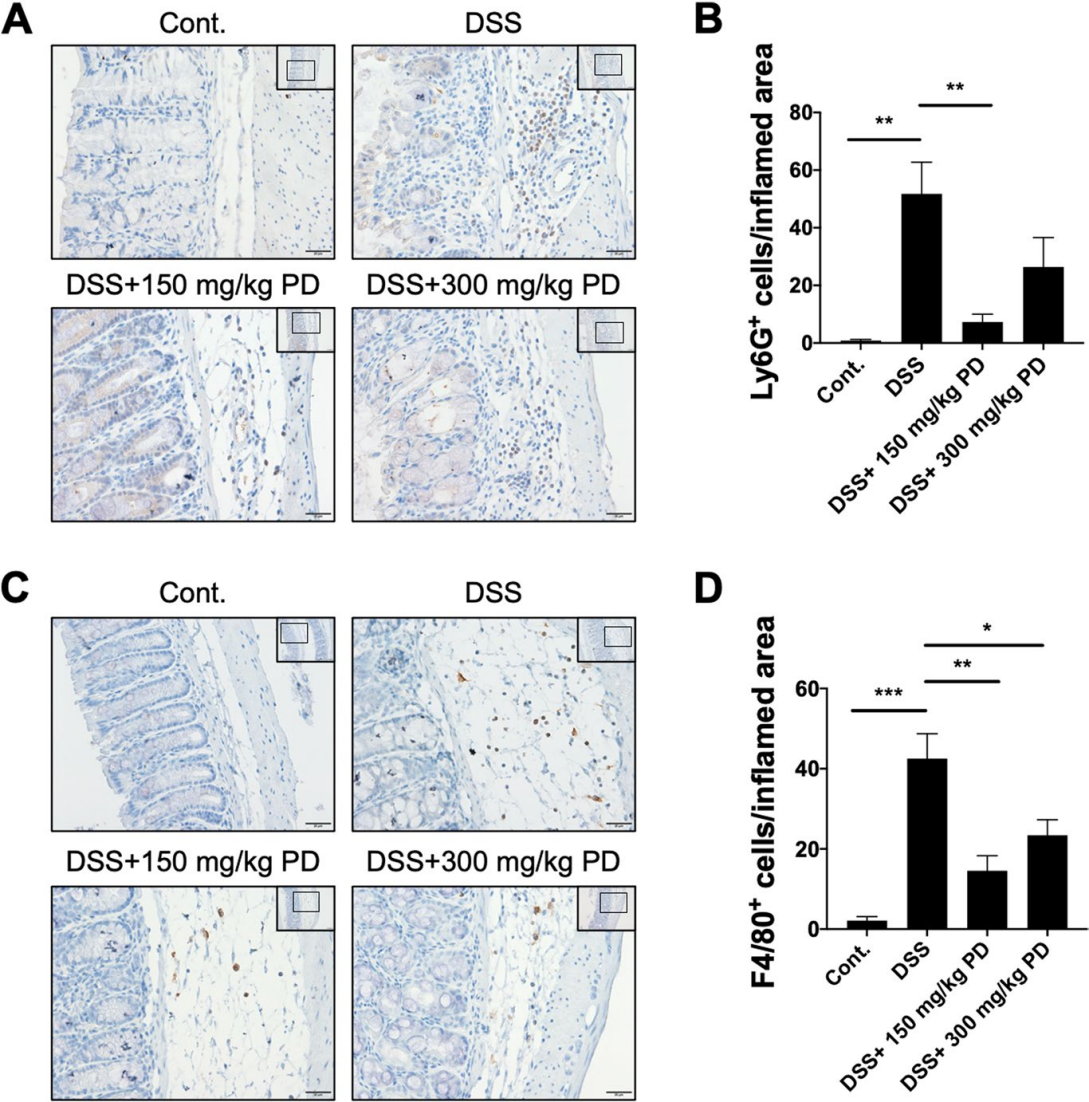
150 mg/kg PD suppresses Ly6G^+^ neutrophil and F4/80^+^ macrophage infiltration in intestinal inflammation. The images of immunohistochemical analysis for the (**A**) anti-Ly6G and (**C**) anti-F4/80 of colonic sections from healthy (Cont.) and DSS-exposed mice treated with or without PD (150 mg/kg and 300 mg/kg). Images were taken at 20X magnification (scale bar: 50 μm, shown in the upper right) and 40X magnification (scale bar: 20 μm). (**B**) Ly6G positive cells and (**D**) F4/80 positive cells were counted in three random inflamed mucosa, respectively, and presented as mean ± SEM, *n* = 5 mice. *p < 0.05, **p < 0.01, ***p < 0.001. PD, Pulsatilla decoction

Since PD reduces the degree of intestinal inflammation in histological analysis (Fig. 1E and F), we were interested in how PD suppresses DSS-induced intestinal inflammation. Thus, we analyzed the number of recruited leukocytes in colonic tissues. Given the essential role of neutrophils in acute tissue injury, we first evaluated the number of neutrophils in infiltrating leukocytes. Ly6G is a cell surface protein predominantly expressed in neutrophils; the immunohistochemistry analysis revealed that compared to control mice, Ly6G^+^ neutrophils were significantly increased in the DSS-induced colitis mouse model and 150 mg/kg PD treatments significantly decreased the DSS-induced infiltration of Ly6G^+^ neutrophils (Figs. 2A and B). As macrophages are usually recruited to the site of inflammation after neutrophils, we next evaluated the number of macrophages in infiltrating leukocytes. Consistent with the neutrophil infiltration, the immunohistochemical (IHC) staining of F4/80, a widely used murine macrophage marker, also displayed a marked infiltration of F4/80^+^ macrophages in the DSS-induced colitis mouse model compared to the control mice (Figs. 2C and D). Furthermore, the infiltration of F4/80^+^ macrophages dramatically declined in inflamed colonic tissues in both 150 mg/kg and 300 mg/kg PD treatments. Collectively, these findings indicate that PD reduces colonic inflammation through the suppression of Ly6G^+^ neutrophil and F4/80^+^ macrophage infiltration.

### PD increases the cytokine level of IL-6 in colonic tissues

**Fig. 3:**
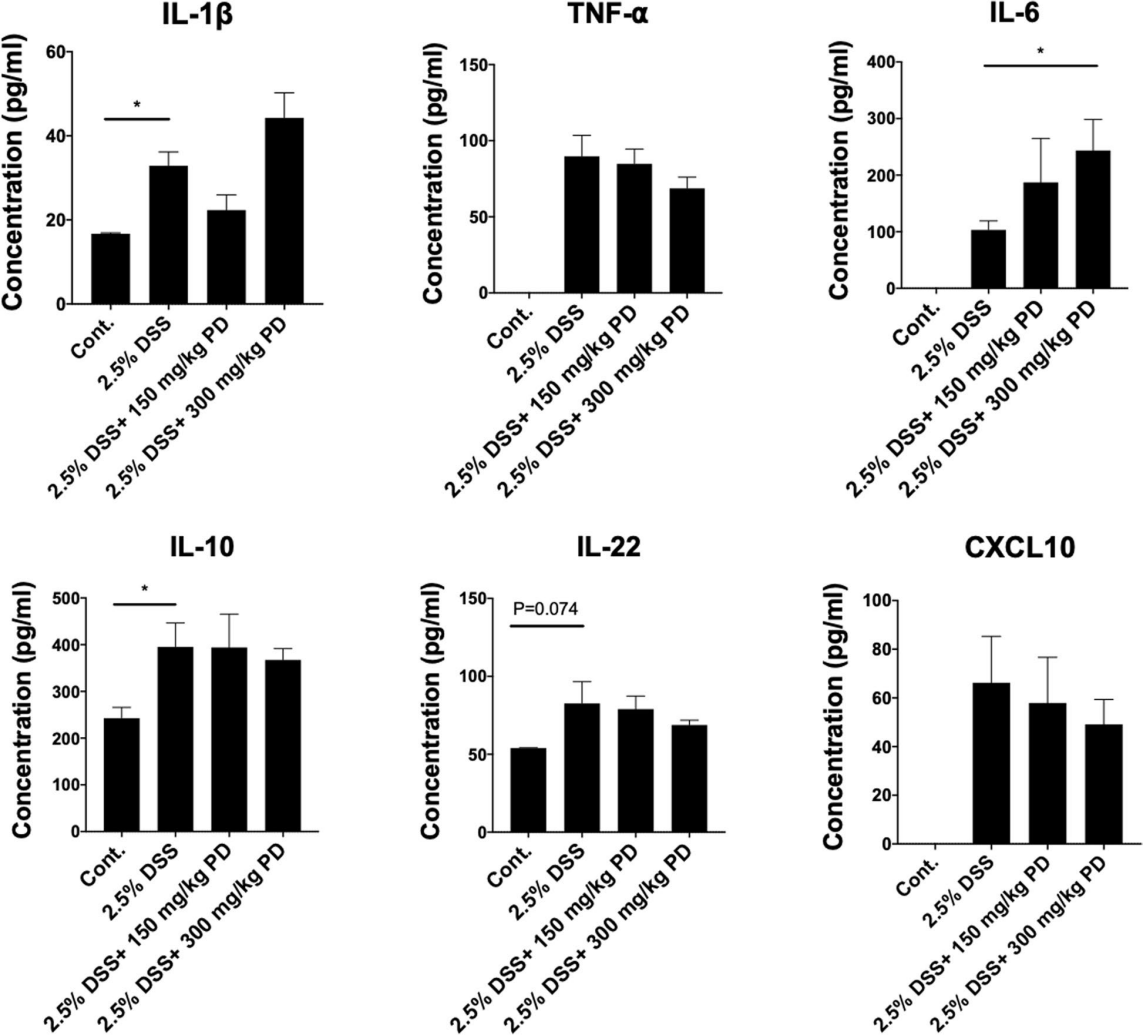
The Multi-Plex analysis demonstrates that 300 mg/kg PD increases the cytokine level of IL-6 in colonic tissues in the DSS-induced colitis mouse model. Colonic tissues from healthy (Cont.) and DSS-exposed mice treated with or without PD (150 mg/kg and 300 mg/kg) were lysed and diluted to a final concentration of 1 mg/ml. The level of mIL-1β, mIL-6, mIL-10, mIL-22, mTNF-α, and mIP-10 in 1 mg/ml colonic lysates were measured through the Multi-Plex immunoassay using Bio-Plex® 200 system. Data are represented as mean ± SEM, *n* = 5 mice. *p < 0.05. PD, Pulsatilla decoction

Cytokines are small secreted signaling proteins that can modulate both pro- and anti-inflammatory immune responses in intestinal inflammation. Based on the observation that PD suppresses immune cell infiltration into colonic tissues, we next asked whether PD can reduce the release of cytokines in DSS-induced colitis mice. Thus, we evaluated the levels of pro- and anti-inflammatory cytokines and chemokine in the colon via Multi-Plex immunoassay. In normal conditions, the levels of cytokines, interleukin (IL)-1β, tumor necrosis factor (TNF)-⍺, IL-6, IL-10, IL-22, and C-X-C motif chemokine 10 (CXCL10), remain low or non-detectable (Fig. 3). However, compared to control, DSS induction significantly induced cytokine and chemokine production in colonic tissues. This phenomenon is consistent with the severe colonic inflammation in DSS-induced colitis mice. Next, we examined the levels of cytokines and chemokines in the colon after PD administration. The results showed that compared to DSS-induced colitis mice, 300 mg/kg PD increased the level of IL-6 but did not significantly alter other cytokine levels in colonic tissues.

### PD does not significantly increase the epithelial proliferation

**Fig. 4:**
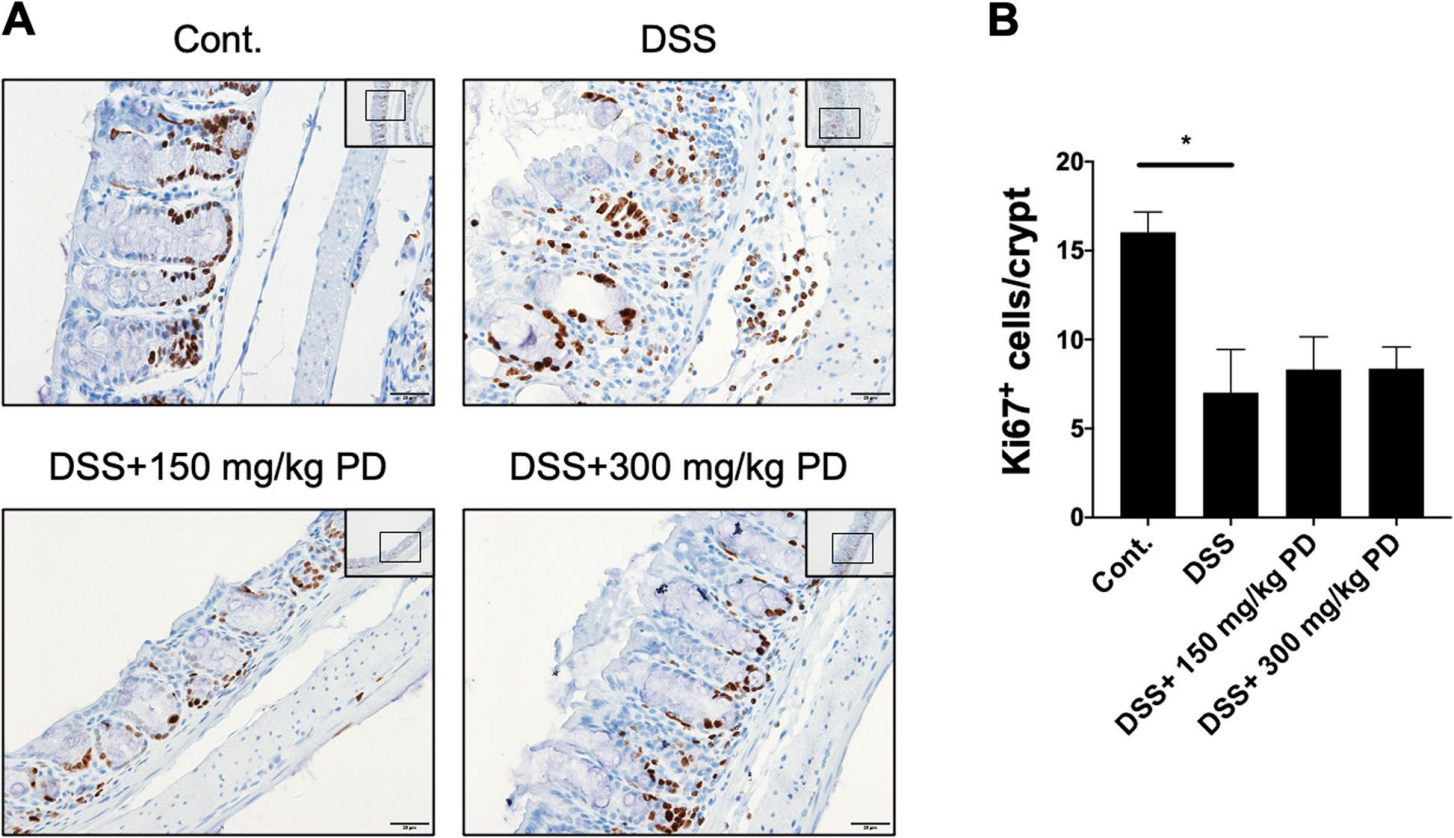
PD does not significantly increase the number of Ki67^+^ proliferating intestinal epithelial cells per crypt in the DSS-induced colitis mouse model. (**A**) The images of immunohistochemical analysis for the anti-Ki67 of colonic sections from healthy (Cont.) and DSS-exposed mice treated with or without PD (150 mg/kg and 300 mg/kg). Images were taken at 20X magnification (scale bar: 50 μm, shown in the upper right) and 40X magnification (scale bar: 20 μm). (**B**) Ki67 positive intestinal epithelial cells were counted in three random areas. In each area, the number of Ki-67 positive intestinal epithelial cells per intestinal crypt was quantified from five random crypts. Data are represented as mean ± SEM, *n* = 5 mice. *p < 0.05. PD, Pulsatilla decoction

Since histological images reveal that the administration of PD suppresses intestinal tissue damage in DSS-induced colitis mice (Fig. 1E), we were interested in whether PD suppresses DSS-induced intestinal damage through enhancing epithelial restoration. Thus, to address this, we performed IHC staining of Ki67 in colonic tissue to examine whether the proliferation of intestinal epithelial cells is altered. The result showed a significant decrease in Ki67^+^ proliferating epithelial cells in DSS-induced colitis mice compared to control mice (Figs. 4A and B), which is consistent with the severe intestinal tissue damage. However, although the IHC images displayed a more intact crypt structure in PD-administered mice, neither 150 mg/kg nor 300 mg/kg PD significantly increased the number of Ki67^+^ proliferating epithelial cells. Therefore, these findings suggest that the reduced DSS-induced intestinal damage in PD treatments does not result from increased Ki67^+^ epithelial proliferation.

### DSS alters the localization of MMP-7 from intestinal epithelial cells to infiltrating immune cells

**Fig. 5:**
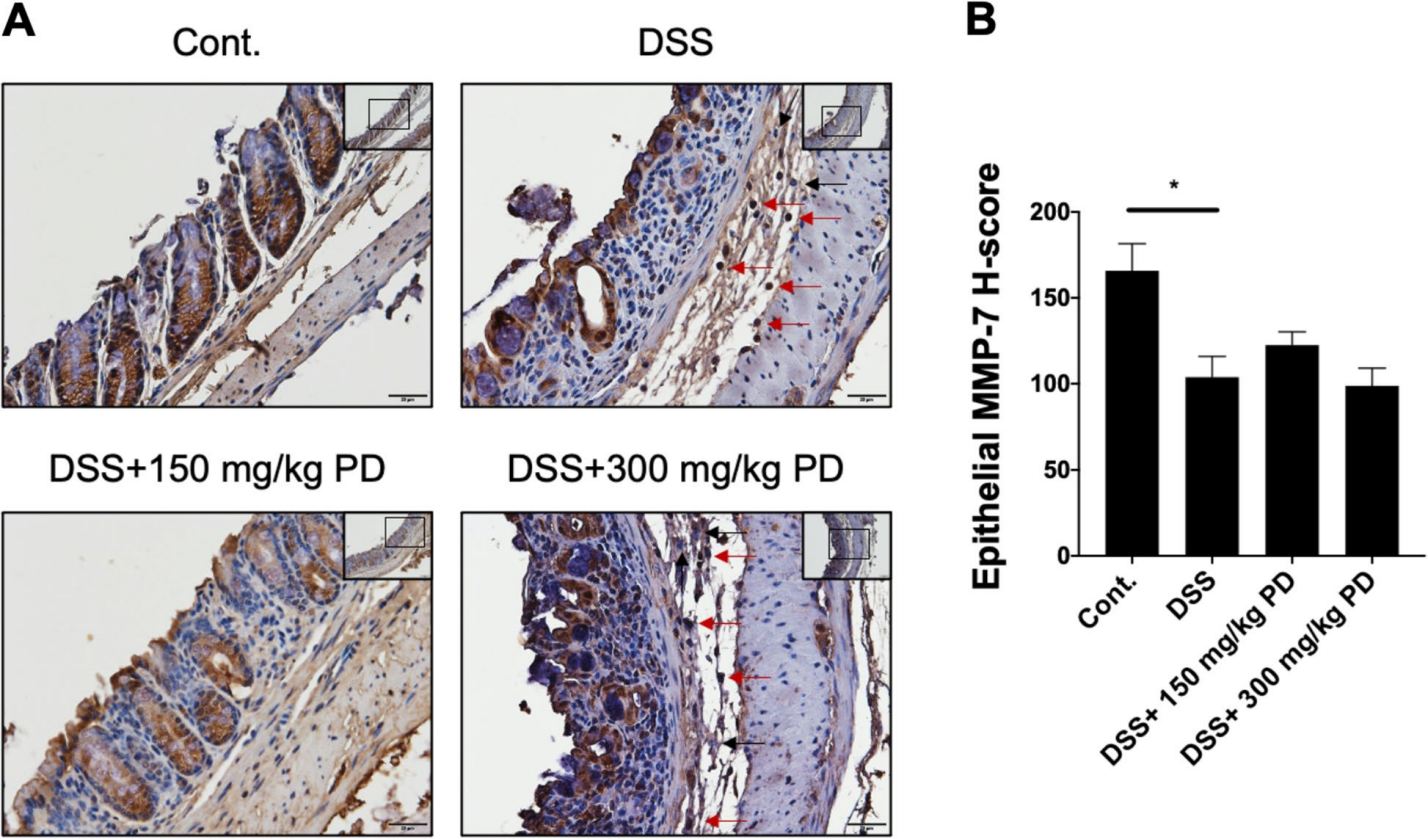
Immunohistochemical analysis of MMP-7 localization in colonic tissues of the DSS-induced colitis mouse model. (**A**) The images of immunohistochemical staining of MMP-7 of colonic sections from healthy (Cont.) and DSS-exposed mice treated with or without PD (150 mg/kg and 300 mg/kg). The MMP-7 positive infiltrating immune cells were indicated by red arrows; the MMP-7 negative infiltrating immune cells were indicated by black arrows. Images were taken at 20X magnification (scale bar: 50 μm, shown in the upper right) and 40X magnification (scale bar: 20 μm). (**B**) The epithelial MMP-7 H-score was measured in three random areas using the DensitoQuant module in 3DHISTECH software. Data are represented as mean ± SEM, *n* = 5 mice. *p < 0.05. PD, Pulsatilla decoction

MMP-7 has shown to be highly expressed in biopsies of UC patients [18, 19]. Since MMP-7 was detected both in colonic epithelial cells and inflammatory cells in colonic sections from UC patients [19–21], we were interested in which cell type could express MMP-7 in the DSS-induced colitis mouse model. The IHC staining of MMP-7 revealed that in control mice, MMP-7 was mainly expressed in colonic epithelial cells. However, in the DSS-induced colitis mouse model, the localization of MMP-7 expression was shifted to the edge of the ulcers and the infiltrating immune cells (Fig. 5A). Moreover, the quantification of the IHC analysis showed a significant reduction in epithelial MMP-7 expression (Fig. 5B). Taken together, these results indicate the association of MMP-7 localization with the severity of colitis, which is consistent with previous clinical findings [20, 21]

### PD suppresses MMP-7^+^ infiltrating immune cells in DSS-induced colitis mouse model

**Fig. 6:**
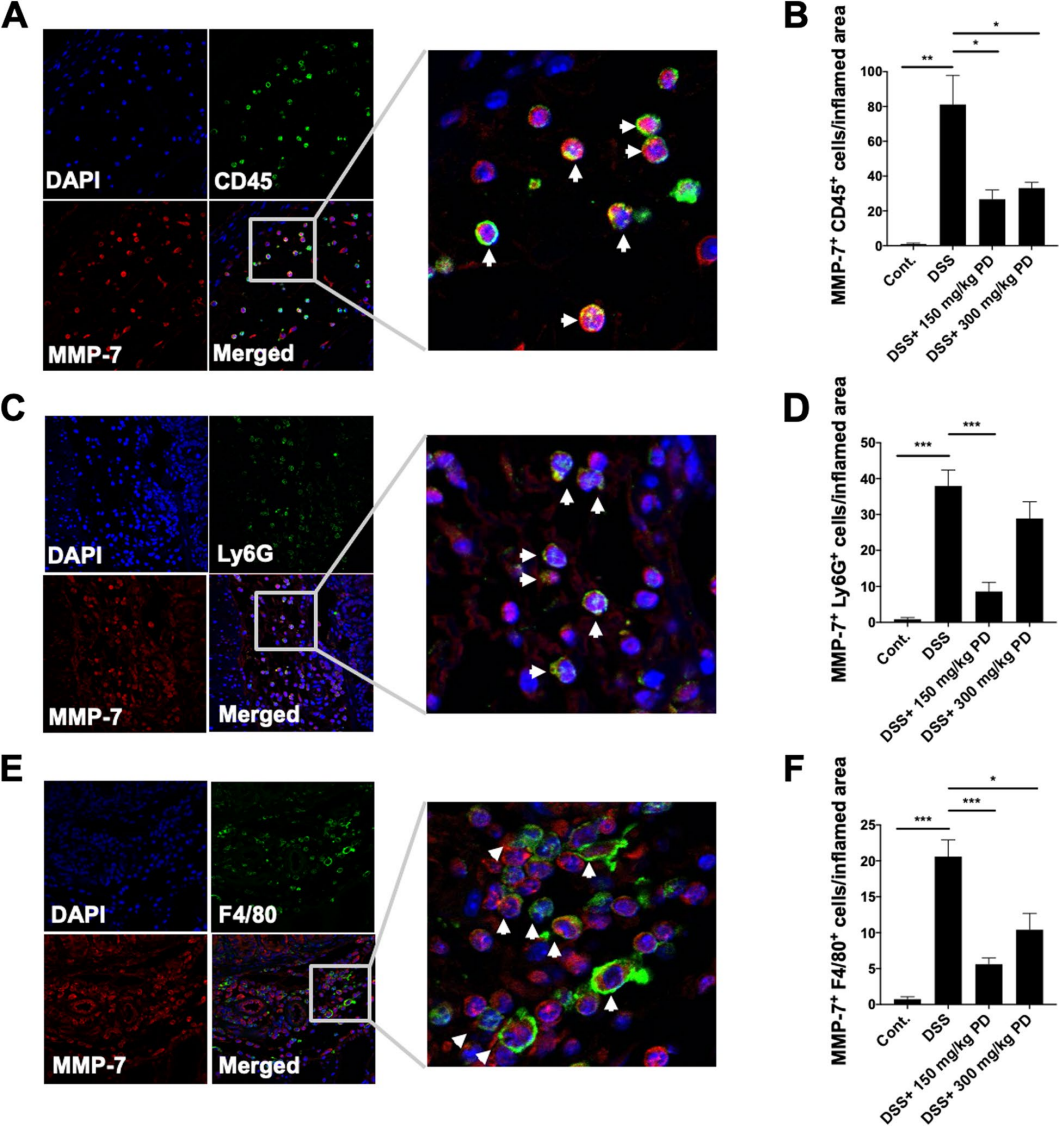
MMP-7 is expressed by the CD45^+^ infiltrating immune cells, Ly6G^+^ neutrophils, and F4/80^+^ macrophages in the DSS-induced colitis mouse model. The immunofluorescence double-staining images of colonic tissues in DSS-induced colitis mice. Expression of (**A**) MMP-7 and CD45, (**C**) MMP-7 and Ly6G, and (**E**) MMP-7 and F4/80 were analyzed by using an anti-MMP-7 antibody (RM7C, red) with an anti-CD45 antibody, anti-Ly6G antibody, or anti-F4/80 antibody (green). Nuclei were labeled in DAPI blue fluorescence. The zoomed-in merged image was shown on the right. The (**B**) MMP-7^+^ CD45^+^ cells, (**D**) MMP-7^+^ Ly6G^+^ cells, and (**F**) MMP-7^+^ F4/80^+^ cells were indicated by white arrows. Images were taken at 40X magnification through LSM 510 confocal microscope

To further confirm that MMP-7 is expressed by the infiltrating immune cells, the immunofluorescence double staining of MMP-7 with CD45, Ly6G, or F4/80 was performed in colonic sections of DSS-induced colitis mice. The result showed that MMP-7 is expressed by CD45^+^ infiltrating immune cells, including Ly6G^+^ neutrophils and F4/80^+^ macrophages, in DSS-induced colitis mice (Figs. 6A, C, and E). Since our previous results show that PD reduces the infiltrating immune cells, we were interested in whether PD also suppresses MMP-7 expression. Therefore, we analyzed the number of MMP-7^+^ CD45^+^ immune cells, MMP-7^+^ Ly6G^+^ neutrophils, and MMP-7^+^ F4/80^+^ macrophages in inflamed colonic tissues. The results showed that PD significantly reduces the MMP-7^+^ Ly6G^+^ neutrophils and MMP-7^+^ F4/80^+^ macrophages among MMP-7^+^ CD45^+^ infiltrating immune cells (Figs. 6B, D, and F), identifying a positive correlation between MMP-7 expression and leukocyte infiltration in DSS-induced intestinal inflammation.

## Discussion

The regulation of intestinal inflammation is believed to critically contribute to the pathogenesis of IBD and the resolution of the intestinal immune response has become an important therapeutic approach in IBD. In the current study, we administered PD, a frequently used TCM, in the DSS-induced colitis mouse model. We demonstrated that PD ameliorates DSS-induced murine colitis through the suppression of intestinal inflammation. This finding is in accordance with reports showing the anti-inflammatory effects exhibited from ingredients of PD [37–40]. In addition, modified PD has been reported to suppress T helper (Th)2-mediated colonic inflammation and restore epithelial integrity in oxazolone-induced murine colitis [42]. Oxazolone-induced colitis is primarily driven by robust IL-13 produced from natural killer T (NKT) cells [46]. However, this phenomenon has not been observed in several clinical studies [47, 48], suggesting oxazolone-induced colitis may not serve as an ideal experimental model. Thus, in our experiments, we have employed a different experimental model, the DSS-induced colitis model, whose inflammatory transcriptomic profiles are similar to UC patients, to analyze the effects of PD in immunomodulation. Our findings revealed that administration of PD in DSS-induced colitis mice suppresses intestinal inflammation resulting in a significant decline in recruiting neutrophils and macrophages into colonic tissues. Since the transcriptomic profiles between DSS-induced colitis mice and UC patients emphasize the conserved pathways related to neutrophil chemotaxis and degranulation as well as the inflammatory immune response [10], our observation of PD reducing leukocyte infiltration provides a new potential mechanism of the action of PD in treating UC.

The colonic infiltration of MMP-7^+^ Ly6G^+^ neutrophils is significantly decreased in the PD-administered DSS-induced colitis mouse model, revealing a positive correlation between the neutrophil infiltration and the expression of MMP-7. Consistent with our finding, neutrophil infiltration has been detected more extensively in biopsies with high MMP-7 expression in patients with rheumatoid arthritis [49]. In addition, MMP-7 has been shown to regulate neutrophil trafficking in tissue injury [13]. MMP-7 sheds syndecan-1, a heparan sulfate proteoglycan present on epithelial cells [14]. The shedding of syndecan-1 subsequently generates a CXCL1/KC chemokine gradient, which leads to the transepithelial neutrophil influx. Moreover, the transepithelial neutrophil influx to sites of injury is impaired in MMP-7^−/−^ mice. Thus, these findings combined with our data demonstrate that MMP-7 is essential for the regulation of neutrophil trafficking in the DSS-induced colitis model and PD suppresses the infiltration of neutrophils by inhibiting MMP-7.

In addition to neutrophil infiltration, the MMP-7^+^ F4/80^+^ macrophage infiltration is significantly decreased in colonic tissues in the PD-administered DSS-induced colitis mouse model. Consistent with our immunofluorescence data, MMP-7 has been found expressed by monocyte/macrophages in several studies [49–52]. Furthermore, MMP-7 has been found to cleave the latent form of the pro-inflammatory cytokine, TNF-α, and release it from macrophages [53]. The release of TNF-α from macrophages induces MMP-3 production, which further generates macrophage chemotaxis and stimulates macrophage infiltration [52]. However, the release of TNF-α from macrophages is abolished in MMP-7^−/−^ mice, showing that MMP-7 is required for macrophage-released TNF-α and subsequent macrophage infiltration. In accordance with the finding, our data showed an increased level of macrophage infiltration, TNF-α production, and MMP-7 activation in DSS-induced colitis mice, while the administration of PD reduces the macrophage infiltration, TNF-α production, and MMP-7 activation in DSS-induced colitis mouse model. Together, these data reveal that PD suppresses the infiltration of macrophages by inhibiting MMP-7 and the production of TNF-α in the DSS-induced colitis mouse model.

Cytokines regulate the cross-talk between intestinal epithelial cells and innate and adaptive immune cells and thus, are important in the pathogenesis of IBD [54]. Through multiplex analysis, we found that DSS induces the release of pro-inflammatory cytokines, IL-1β and TNF-α, which facilitates the inflammation in the DSS-induced colitis mouse model. In addition, the anti-inflammatory cytokines, IL-10 and IL-22, are also increased in DSS- induced colitis, suggesting that negative feedback is activated to limit the progress of inflammation. Although the administration of PD does not alter most cytokine levels, we did observe a significant increase in IL-6 production in 300 mg/kg PD treatments in DSS- induced colitis mouse model. IL-6 secreted by intraepithelial lymphocytes has been shown to increase tight junction protein expression and mucus secretion, which promotes the integrity of the epithelial barrier [55]. The colon length is an indicator of intestinal damage in the DSS-induced colitis mouse model [44]. A significant colon length shortening indicates the presence of severe intestinal damage, while the reduced colon length shortening indicates amelioration of intestinal damage. Consistent with the elevated IL-6 production, 300 mg/kg PD significantly reduces the colon length shortening in the DSS-induced colitis mouse model. Therefore, our findings suggest that PD increases the level of IL-6 to enhance epithelial restoration in the DSS-induced colitis mouse model.

## Conclusions

**Fig. 7:**
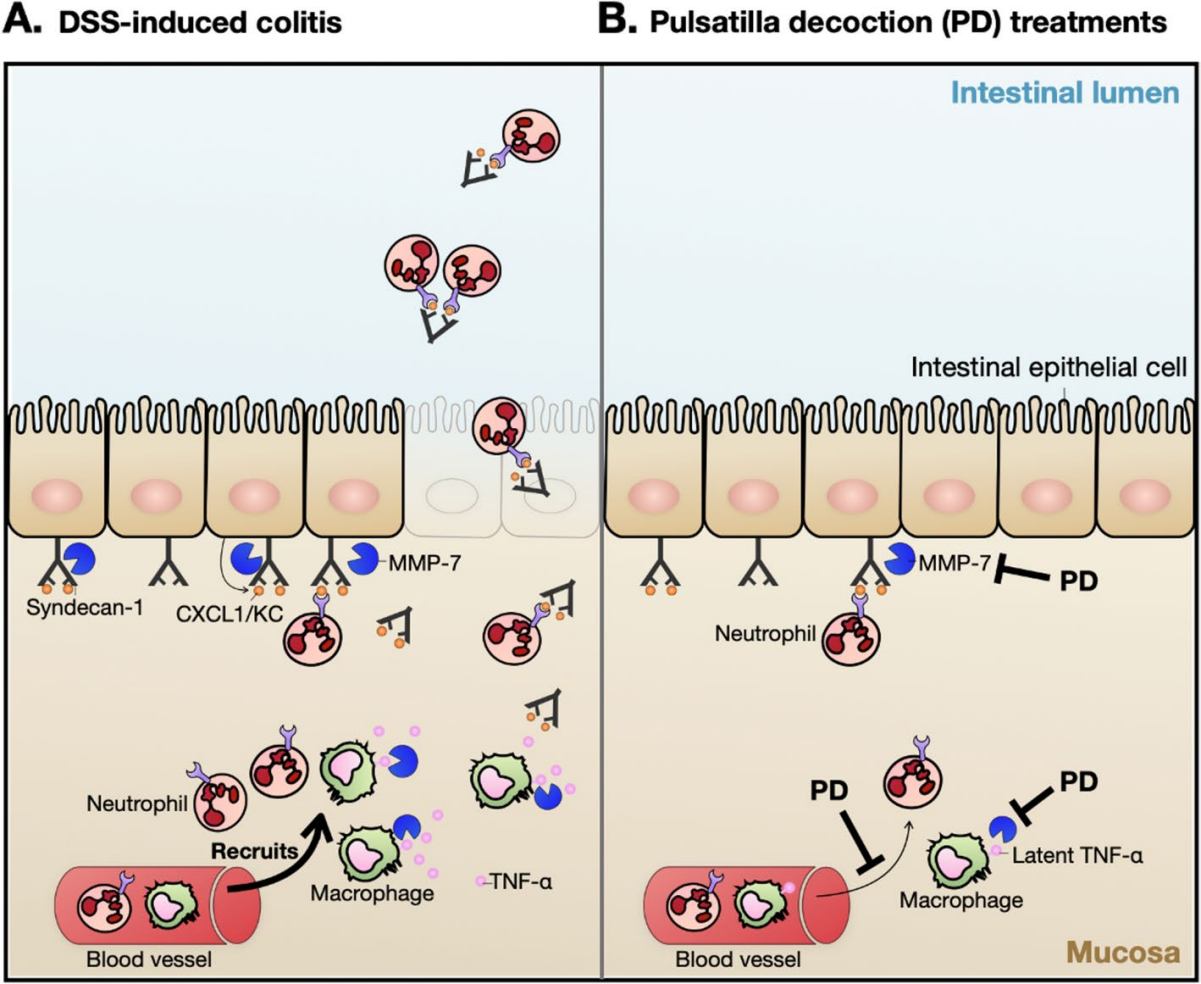
MMP-7 participates in the molecular mechanism underlying PD in the DSS-induced colitis mouse model. (**A**) In the DSS-induced colitis mouse model, MMP-7 induces the infiltration of neutrophils and macrophages by generating a CXCL1/KC chemokine gradient and release of TNF-α from macrophages. (**B**) The administration of PD significantly inhibits the activities of MMP-7 and thereby suppresses the infiltration of neutrophils and macrophages as well as the intestinal inflammation in the DSS-induced colitis mouse model

PD attenuates ulcerative colitis in the DSS-induced colitis mouse model. PD suppresses intestinal inflammation, with a marked decrease in colonic infiltration of innate immune cells, through decreasing MMP-7 expression. MMP-7 plays an important role in intestinal innate immune regulation (Fig. 7).

## Supplementary Information

**Additional file 1.**

## Data Availability

Not applicable.

## Abbreviations

UC: Ulcerative colitis
DSS: Dextran sulfate sodium
PD: Pulsatilla decoction
MMP-7: Matrix metalloproteinase-7
IBD: Inflammatory bowel disease
CD: Crohn’s disease
IHC: Immunohistochemical
5-ASA: 5-Aminosalicylic acid
TCM: Traditional Chinese medicine
DAI: Disease activity index
H&E: Hematoxylin and eosin
