# Supplementary material for: Pulsatilla decoction suppresses matrix metalloproteinase-7-mediated leukocyte recruitment in dextran sulfate sodium-induced colitis mouse model
Source: BMC Complement Med Ther. 2022 Aug 6;22:211. doi: 10.1186/s12906-022-03696-w (PMC9356479; doi:10.1186/s12906-022-03696-w)

**Supplementary material**

**Method**

**Liquid chromatography-mass spectrometry/mass spectrometry (LC-MS/MS) Analysis**

The dried residue of Pulsatilla decoction (PD) was dissolved into 50% methanol to prepare a solution with a concentration of 1 mg/mL for LC-MS/MS analysis. LC-MS/MS analysis conditions were as follows. The high-performance liquid chromatography (HPLC) separation in LC-MS/MS was carried out on an Agilent zorbax eclipse XDB-C8 column (5 μm, 150 mm x 4.6 mm). 5 μL of PD solution was injected into the LC column. A linear gradient at a flow rate of 0.25 mL/min was used to elute the compounds in the PD. The mobile phase consisted of water containing 0.1% (v/v) formic acid (A), and acetonitrile containing 0.1% (v/v) formic acid (B), with a gradient of B from 10% to 70% in the first 3 mins, then up to 100% B over 2 mins, held at 100% B for another 5 mins, then down to 10% B over 0.1 min, and held at 10% B for another 4.9 mins at a flow rate of 0.25 mL/min. The HPLC was performed on the Agilent 1260 HPLC system (Santa Clara, USA) equipped with a G1312B Binary Pump and a G1367E HiP ALS Autosampler. AB Sciex Instruments QTRAP 5500 with a Turbo V Ion Source was used as a mass spectrometer suitable for LC-MS/MS experiments. The ion source was operated at 5500 V in positive ion mode and -4500 V in negative ion mode. Nitrogen was used as a nebulizing (60 psi) and heating gas (50 psi). Multiple Reaction Monitor and Selected Ion Monitor scan types were used. Betaine anhydrous, Loganin, Sesamin, 5-Hydroxymethylfurfural, β-Carotene, Gallic acid, Protocatechuic acid, Chlorogenic acid, Ferulic acid, and Ursolic acid were used as the standards. Q1 and Q3 mass of these standards were used as the references. Data were processed with Analyst 1.5 software (Foster City, USA). The method of Multiple Point External Standard was used for the quantification.

**Result**

**Supplemental Table 1. Compounds and their concentrations (μg/mg) identified in
Pulsatilla decoction by LC-MS/MS Analysis in positive and negative ion mode**

| **Compounds** | **Positive ion mode** | **Negative ion mode** |
| --- | --- | --- |
| Betaine anhydrous | 0.377 | - |
| 5-Hydroxymethylfurfural | 0.353 | - |
| Loganin | 0.137 | - |
| Sesamin | 3.460 | - |
| β-Carotene | 0.246 | - |
| Gallic acid | - | 0.032 |
| Protocatechuic acid | - | 0.133 |
| Chlorogenic acid | - | 2.720 |
| Ferulic acid | - | 0.499 |
| Ursolic acid | - | 0.165 |

Supplemental Figure 1. Representative image of immunofluorescence staining colonic section of each experimental group in Figure 7. The section used for this experiment is indicated by a red circle.


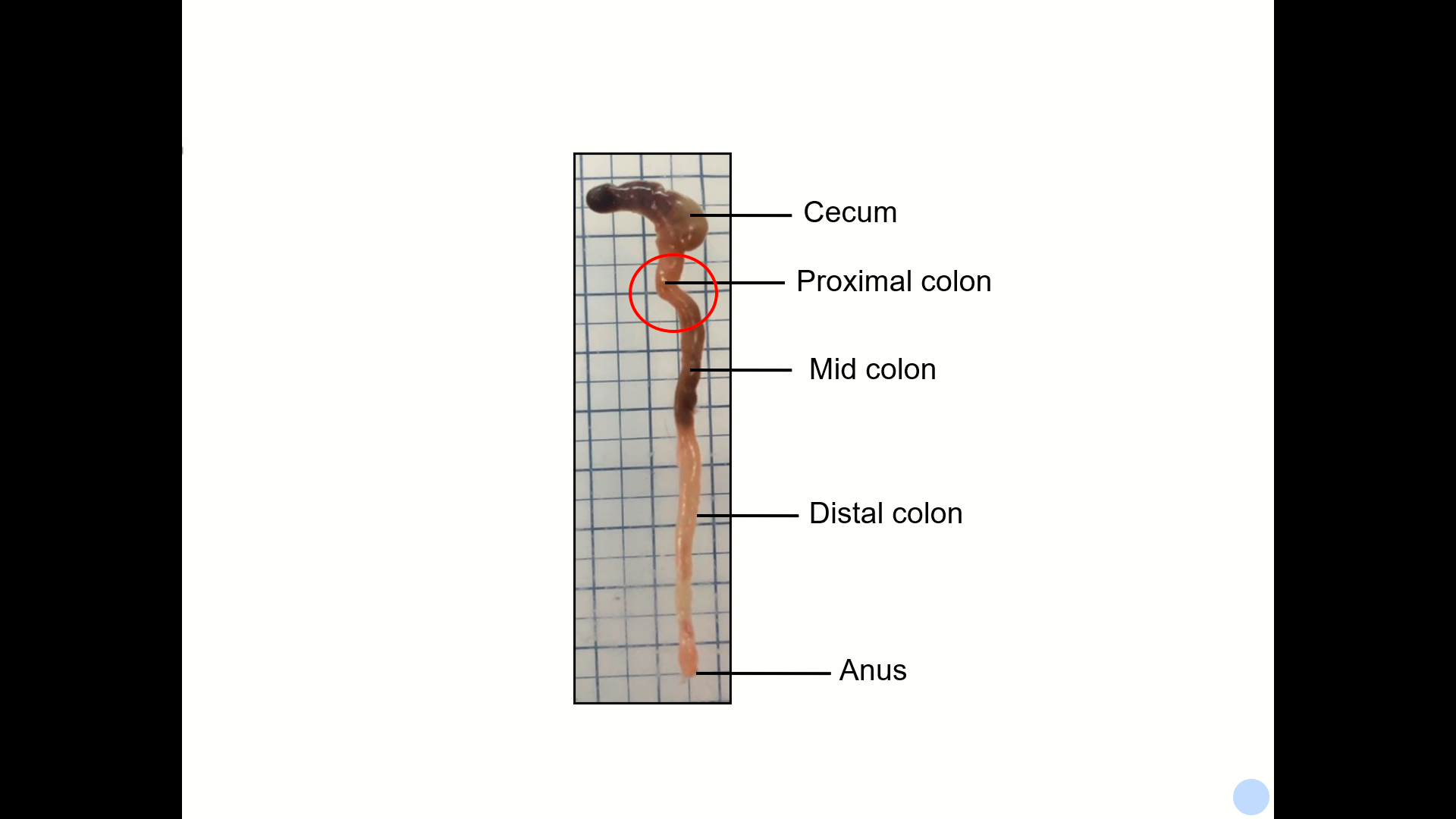

Supplement: Supplementary file 1 — Additional file 1. [file 12906_2022_3696_MOESM1_ESM.docx]
